# Association between DNA methylation variability and self-reported exposure to heavy metals

**DOI:** 10.1038/s41598-022-13892-w

**Published:** 2022-06-22

**Authors:** Anna Freydenzon, Marta F. Nabais, Tian Lin, Kelly L. Williams, Leanne Wallace, Anjali K. Henders, Ian P. Blair, Naomi R. Wray, Roger Pamphlett, Allan F. McRae

**Affiliations:** 1 Institute for Molecular Bioscience, The University of Queensland, Brisbane, QLD 4072 Australia; 2 University of Exeter Medical School, Exeter, EX2 5DW Devon UK; 3 Centre for Motor Neuron Disease Research, Macquarie University, Exeter, NSW 2109 Australia; 4 Queensland Brain Institute, The University of Queensland, Brisbane, QLD 4072 Australia; 5 Brain and Mind Centre, The University of Sydney, Sydney, NSW 2050 Australia

**Keywords:** Genetics, Epigenetics

## Abstract

Individuals encounter varying environmental exposures throughout their lifetimes. Some exposures such as smoking are readily observed and have high personal recall; others are more indirect or sporadic and might only be inferred from long occupational histories or lifestyles. We evaluated the utility of using lifetime-long self-reported exposures for identifying differential methylation in an amyotrophic lateral sclerosis cases-control cohort of 855 individuals. Individuals submitted paper-based surveys on exposure and occupational histories as well as whole blood samples. Genome-wide DNA methylation levels were quantified using the Illumina Infinium Human Methylation450 array. We analyzed 15 environmental exposures using the OSCA software linear and MOA models, where we regressed exposures individually by methylation adjusted for batch effects and disease status as well as predicted scores for age, sex, cell count, and smoking status. We also regressed on the first principal components on clustered environmental exposures to detect DNA methylation changes associated with a more generalised definition of environmental exposure. Five DNA methylation probes across three environmental exposures (cadmium, mercury and metalwork) were significantly associated using the MOA models and seven through the linear models, with one additionally across a principal component representing chemical exposures. Methylome-wide significance for four of these markers was driven by extreme hyper/hypo-methylation in small numbers of individuals. The results indicate the potential for using self-reported exposure histories in detecting DNA methylation changes in response to the environment, but also highlight the confounded nature of environmental exposure in cohort studies.

## Introduction

DNA methylation (DNAm) is the addition of a methyl group to DNA, most commonly at CpG dineculotide sites in mammals. DNAm provides an epigenetic mechanism for the regulation of transcription, playing a vital role in cellular differentiation, X chromosome inactivation and maintaining genomic stability^1^. Furthermore, DNAm is influenced by both innate effects (i.e., ageing and cell type) as well as environmental exposures which further interact with each other^2^.

If predictable DNAm changes occur as a consequence of environmental exposure then DNAm measures can be used to construct a proxy measure for exposure in the absence of direct measurement of the exposure. For example, cigarette smoking results in substantial changes in DNAm at hundreds of sites across the genome^3^, with the changes in a proportion of these sites remaining long after smoking cessation^4^. The methylation at these CpG sites can be used to predict both current and prior smoking status, with the potential to validate or replace inaccurate measures such as self-reporting^5^. Individuals whose exposure to cigarette smoking is secondary (e.g., through living with a spouse who smokes or being raised in a smoking household) can be identified from DNA methylation measures, whereas they would otherwise self-report as a non-smoker^6^. This approach of identifying historical exposures from their effects on DNAm has a range of potential uses, particularly in disease epidemiology, because relevant exposures may be difficult to quantify or are unknown to the study participant.

Environmental toxicants are suspected to have a role in the etiology of some neurodegenerative disorders^7,8^. For example, lead and aluminium exposure accelerates the accumulation of amyloid-peptide deposits related to Alzheimer’s disease^8,9^ and manganese can trigger Parkinsonism^10^. Amyotrophic lateral sclerosis (ALS) is a neurodegenerative disease characterized by upper and lower motor neuron dysfunction with little variation being explained by known heritable genetic factors, and is the most prevalent form of all motor neurone diseases (MND)^11^. ALS has been reported to be associated with a large range of environmental factors, including heavy metals such as lead^7,12^, cycad genotoxins^13^, and unknown causative agents associated with military service^14^. However, the replication of these association studies has been poor^15^, mostly due to difficulties in quantifying individual exposure due to a lack of standardised methodology.

In the current study, methylation-wide association studies (MWAS) were performed on a range of self-reported environmental exposures as potential ALS risk factors. While the study participants were ALS patients and non-ALS controls, the focus was on the exposures using ALS status as a covariate. Several associations between DNA methylation and metal exposures were identified, which highlights the confounded nature of assessing many exposures.

## Materials

### Sample collection and participant inclusion

ALS patients (cases) and controls were recruited between April 2000 to June 2011 as part of The Australian MND DNA Bank from the University of Sydney^16^. Cases were white Australians older than 25 years recruited from around Australia via state-based MND association newsletters, which were classified as having ALS based on verification by a neurologist^17^. Controls had no history of neuromuscular disease, and were recruited as friends or spouses of case participants, or community volunteers. Exclusion criteria were any family history of ALS and slowly progressive disease, defined as survival for greater than 10 years after diagnosis. Participants provided a blood sample for DNA analysis.

Participants were asked to complete a 6-page paper questionnaire. This questionnaire included short-form, multiple-choice and yes/no questions under the following categories: General, lifetime residences, lifetime travel, family illnesses, lifetime employment, exposure to chemicals or toxins, injuries, physical exercise, personal habits, past illnesses, and medication*.* A total of 855 genetically confirmed unrelated individuals (438 ALS cases and 417 controls) were included in the analysis. The age and sex distributions of the groups are reported in Table S1, with mean age and percentage female being 59 years and 54% for controls, 62 years and 38% for cases, respectively.

Collection of DNA and questionnaire information was approved by the Human Research Ethics Committee of the Sydney South West Area Health Service. All DNA data generation and analyses were approved by the University of Queensland Human Research Ethics Committee. All research was performed in accordance with relevant regulations and guidelines, and informed consent was obtained from all participants.

### Evaluation of environmental exposures reported in questionnaire

**Figure 1:**
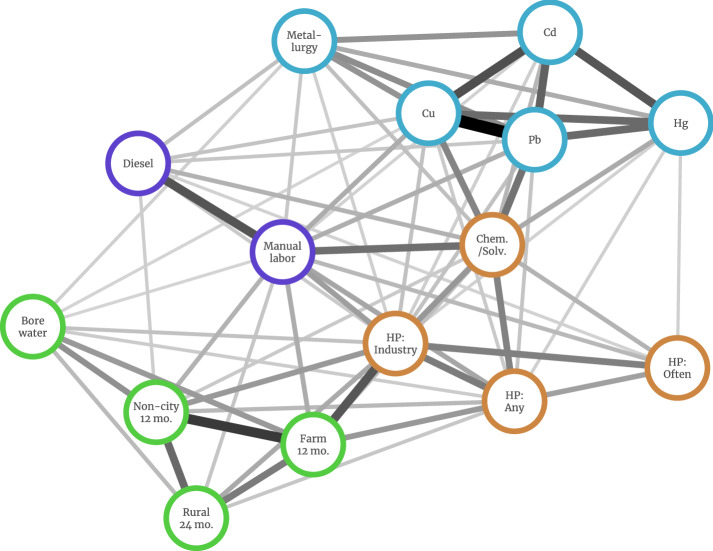
Correlation network of tested environmental phenotypes. Edges shown have $p < 0.05$ after Holm correction for multiple testing. Edge weight and repulsion are based on strength of correlation, which are all positive. Nodes are colored based on final cluster allocation: Mine (blue), Chem (orange), Farm (green) and 'blue collar' Work (purple) exposures. HP: Herbicide/pesticide. Chem/Solv: Other chemicals or solvents. Cu: Copper. Cd: Cadmium. Hg: Mercury. Pb: Lead. For variables suffixed with *n* mo., they qualify living in that locale within the last *n* months.

Figure S1 delineates the steps of the analysis as a flowchart. A total of 15 binary-response (yes/no) questions relating to environmental exposures were selected from the questionnaire. Unmarked fields were interpreted as an implied ‘no’, since participants often responded only in the case of exposure^18^. To consolidate highly-related exposures, we used the hierarchical clustering algorithm within the R Clustovar package^19^. Cluster numbers from 2 to 15 were tested, with the cluster stability (Figure S2) and the variance explained by the first principal component of the clusters (Figure S3) used to determine the optimal grouping. The principal component analyses of the final clusters are reported in Figure S4. All 15 individual binary variables were exported as outcome variables for the methylation profiling, as well as the first principal component of each of the four final clusters (Fig. 1). Self-reported current smoking status was used as a validation variable due to its established status as an exposure that causes methylation changes.

### Array and DNA methylation processing

Measurement and normalisation of DNAm is described elsewhere^20^. Briefly, typing for $468,521$ DNAmsites was conducted using the Infinium HumanMethylation450 BeadChip array, following the standard Infinium HD Methylation protocol. The R Bioconductor package meffil^21^ was used for initial processing, normalization and quality control following filtering thresholds, as detailed previously^20^. The mean and minimum bisulfite II conversion efficiencies were 12.47 and 11.51, respectively, falling above the suggested manufacturer threshold of 1 for successful conversion and higher than the majority of values reported in a metanalysis of 80 datasets^22^. Cell-type proportions were estimated based on the GSE35069 blood methylome profile through the meffil implementation of the Houseman algorithm^23^. Probes which were sex-chromosome linked, non-uniquely hybridized, or with internal SNPs adjacent to the 3’ end were removed according to previously recommended criteria^24^. In addition, probes with low variability (SD < 0.02) were removed. After quality control, a total of $m = 156,874$ DNAm sites remained.

Probe measures were precorrected with predicted scores for sex, age^25^, smoking^26^ (omitted for the smoking analysis), and for predicted cell types B, CD4-T, CD8-T, NK-T, monocytes and neutrophils to address bias in cell type composition in DNAm^23^. To account for batch effects, row and column position were added to the models, with slide (beadchip) as the sole random effect. The residuals of these linear mixed-effects models, generated through the R lme4 package^27^, were taken forward for the analyses.

### Methylome-wide association analysis

Omics-data-based Complex Trait Analysis (OSCA) software^28^ was used to conduct the methylome-wide association analysis for the exposure variables. The effects of environmental exposures on DNA methylation were tested using several approaches. First, simple linear regressions with each exposure or exposure PC considered as the dependent variable regressed on a single probe fitting ALS status (a binary value denoting control or case) as a covariate. Secondly, we used OSCA’s mixed model approach MLM-based omic association, or MOA using ALS as a covariate, but with a random effect added to the linear model representing genome-wide DNAm level of an individual. The variance/covariance structure of the random effect is described by the omic-data-based relationship matrix (ORM), in which off-diagonal elements capture the genome-wide similarity of DNA methylation values between pairs of individuals. This approach has been shown in the original publication to be robust to confounding of probes across the the genome. When possible, we also ran the highly conservative multi-component MLM-based omic association excluding the target (MOMENT) method which used two random effects in the model constructed from two sets of probes grouped based on genome-wide significance, omitting those within 50 kb of the tested probe. Applying the MOMENT method is only possible or relevant when a set or locally high correlated probe associations is detected^29^. We stringently accounted for multiple testing across CpGs by using a Bonferroni correction for epigenome-wide significance threshold ($0.05/m = 3.19* 10^{ - 7}$ for the *m* DNAm sites tested). No additional multiple testing corrections were made for the 15 exposures due to their extensive correlation structure.

To verify the robustness of the significance tests, 1000 permutations of the MOA model were performed for phenotypes with epigenome-wide significant probes, by random reassignment of the phenotype (with or without replacement) to the DNAm data. The number of epigenome-wide significant probes for each permutation, as well as the p-values of previously detected probes, was extracted from each model.

A differentially methylated region (DMR) analysis was applied to the complete results of all MOA models using comb-p to determine whether the epigenome-wide differentially methylated probes belong to a DMR of probes methylated in the same direction. This software’s full pipeline aggregates the p-values of adjacent probes to calculate an autocorrelation factor, combines p-values through the modified Stouffer-Liptak-Kechris (SLK) correction and finally returns region p-values with and without the multiple testing Sidak correction^30^. The parameters for seed threshold was set to 0.05 and window distance to 750 base pairs as the best performing settings according to a previously published benchmark study^31^.

## Results

Endorsement counts of environmental exposures included in the analyses are reported in Table 1. Across these 15 variables, the median response rate was 96% with a minimum of 71%. These variables all had high proportions of nonresponse which were interpreted as ‘no’ responses, given that other parts of the questionnaire had been fully completed.

**Table 1:** Proportion of cases and controls reporting exposure to each phenotypic variables. Individual exposures were tested using Fishers Exact Test. The difference in means of the first principal component of each cluster were tested using a two-sided student's t-test. HP: herbicide/pesticide, OR: odds ratio, PC1: principal component 1.

	Control (n = 417)			ALS (n = 438)				95% CI		
	*n*	%	Mean	*n*	%	Mean	*p*	OR	Low	High
Metal PC1			− 0.17			0.16	0.002		− 0.55	− 0.12
Cadmium	13	3.1		27	6.2		0.036	2.04	1	4.37
Metallurgy	22	5.3		40	9.1		0.034	1.8	1.02	3.25
Lead	40	9.6		78	17.8		< 0.001	2.04	1.34	3.15
Mercury	21	5		30	6.8		0.312	1.39	0.75	2.59
Copper	45	10.8		67	15.3		0.054	1.49	0.98	2.29
Chemical PC1			− 0.27			0.26	< 0.001		− 0.71	− 0.35
Chemicals/solvents	146	35		238	54.3		< 0.001	2.21	1.66	2.94
Any HP use	224	53.7		290	66.2		< 0.001	1.69	1.27	2.25
Regular HP use	24	5.8		47	10.7		0.009	1.97	1.15	3.43
Industrial HP use	43	10.3		83	18.9		< 0.001	2.03	1.35	3.1
Farm PC1			− 0.2			0.19	< 0.001		-0.57	-0.2
Bore water	57	13.7		80	18.3		0.076	1.41	0.96	2.08
Rural ≥ 24 months	62	14.9		110	25.1		< 0.001	1.92	1.34	2.76
Farm ≥ 12 months	93	22.3		130	29.7		0.016	1.47	1.07	2.03
Non-city ≥ 12 months	202	48.4		260	59.4		0.002	1.55	1.18	2.06
Blue Collar PC1			− 0.21			0.2	< 0.001		− 0.56	− 0.24
Diesel	98	23.5		157	35.8		< 0.001	1.82	1.33	2.48
Manual labor	170	40.8		244	55.7		< 0.001	1.83	1.38	2.42
Smoker	50	12		32	7.3		0.027	0.58	0.35	0.94

### Clustering of environmental exposures

**Figure 2:**
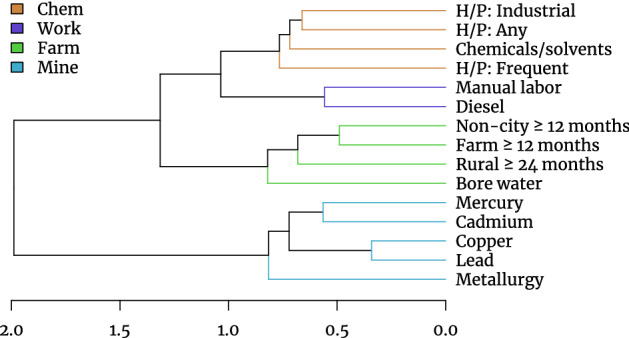
Hierarchical clustering of exposure measures. Exposures were separated into $k = 4$ clusters (coloured) based on cluster stability over 100 bootstrap samples.

The clustering analysis of the questionaire response items generated four partitions, with this choice being justified by cluster stability analyses, and the variance explained by the principal components of the partitions (Figures S1-3). The correlation network (Fig. 1) and the clustering dendrogram (Fig. 2) illustrate these final four partitions. The cluster with the largest height or homogeneity, with 5 constituent exposures, represents questions that can be broadly summarized as metal exposures. These relate to either exposure to named metals (cadmium, lead, copper or mercury) or working in occupations that involve metallurgy, either handling metallic ores or creating metals. We labelled this partition ‘Metal’. The second largest partition represents indicators for living or working in rural/farm areas. In addition to survey questions relating to residence outside of urban areas, the exposure to and use of bore water was included in this cluster which we refer to as ‘Farm’. The third partition consists of a grouping of herbicide/pesticide (HP) exposures (Intermittent domestic, Regular domestic or Industrial use) as well as exposure to other chemicals or solvents. The fourth partition was “blue collar” or “manual” work, with the inclusion of diesel exposure, or manual labor roles obtained from occupational reporting. We refer to these two groups as Chemical (abbreviated Chem) and Work, respectively.

The grouping of manual labor and exposure to diesel fuel in the fourth partition may reflect the use of occupation-reporting fields to identify diesel exposure. Diesel exposure was not explicitly indicated on the questionnaire, but we interpreted likely diesel exposure as having worked within a set of ANZCO/ISCO occupation codes (Table S2) formerly established as high users of diesel fuel through this cohort by Pamphlett and Rikard-Bell^32^. The classification of former or current work in manual labor was directly indicated for each listed occupation on the questionnaire form.

The correlation network (Fig. 1) shows a degree of correlation between all exposures. In the case of the metal exposures, the proportion of cases and controls who have reported exposure is relatively small ($\le 17.8\%$, Table 1). The first principal component generated from each of the four partitions was then used as a continuous variable representative of its correlated constituents, which are otherwise binary. The proportion of variance explained by each are 51% for Mine, 46% for Chem, 50% for Farm, and 72% for Work (Figures S2-3).

### Methylome-wide association analyses

We benchmarked our dataset for the ability to detect effects on DNAm from environmental exposures by using the exposure variable of self-reported smoking. Within our cohort of 855 participants, 50 of 438 ALS cases and 32 of 417 controls report themselves as current smokers (Table 1). Linear and MOA analyses identify $m = 212$ and $m = 74$ smoking-associated probes, respectively (minimum association p-value of 3.19e − 7). The Pearson correlation of smoking probe effect sizes compared to those published previously^33^ (for the current versus never-smoker contrast) was 0.64 and 0.68 for the linear and MOA and models respectively, comparing probes FDR < 0.05 in the reference data ($m = 18,760$). The MOMENT analysis identified two probes associated with *AHRR* that have been shown to explain the largest proportion of variation in smoking status and lung cancer risk^34^, as well as an unannotated intergenic region. These *AHRR* probes (cg05575921 and cg26703534) are hypomethylated in smokers, while the significance of the unannotated region appears to be driven by hypomethylated outliers in the nonsmoking group (Figure S5). The stringency of MOMENT compared to the linear and MOA methods is visualized within Figures S5-6. The top 20 probes detected in the MOA analysis are reported in Figure S4. These results demonstrate that our cohort size is well-powered to detect these known effects, with a smoking rate of ~ 10%.

In contrast to self-reported smoking status, there were fewer associations detected across the 15 individual binary environmental exposure variables. A total of 7 epigenome-wide significant sig- natures were detected by linear regression across exposures for cadmium, mercury, and metallurgy (Table 2). Of these associated CpG sites, cg10071091 (annotated to *KSR2*) for metallurgy was the only one that did not pass the significance threshold via MOA. Likewise, the Chem PC1 continuous variable was associated with cg09369863 (annotated to *LINGO1*) through the linear method, though not MOA. The peaks of co-located inflated loci as seen in the smoking MWAS using MOA (Figure S7b) are not present in any of the environmental MWAS with associated CpGs (Figure S8) and as such the gain in stringency from MOMENT^28^ is not needed. The Q-Q plots of each of these models are reported in Figure S9, with little difference between the linear and MOA methods. We consider the small deflation in significance from the linear to the MOA models as indicative that the observed associations in the linear models were not solely influenced by the confounding structure within local and distal probes, and regard probes detected by both methods as less likely to be false positives.

**Table 2:** Significant probe associations ($p < 3.19e^{ - 7}$) detected across the 15 environmental exposure traits studied, as well as the 4 cluster PC1s.

Variable	Probe	Genes	Chr	Position	Method	β^1^	SE	*p*
Cadmium	cg03085637	*GNRHR2*;	1	145,918,458	Linear	1.86	0.29	9.4 × 10^–11^
		*PEX11B*			MOA	0.05	0.01	7.5 × 10^–11^
	cg21124714	*P2RY6*	11	73,272,052	Linear	− 1.54	0.3	2.6 × 10^–7^
					MOA	− 0.04	0.01	1.8 × 10^–7^
	cg16655883	*ZFR2*	19	3,868,626	Linear	1.18	0.23	2.7 × 10^–7^
					MOA	0.04	0.01	1.8 × 10^–7^
Chem PC1	cg09369863^2^	*LINGO1*	15	77,820,505	Linear	11.68	2.25	2.1 × 10^–7^
					MOA	0.24	0.05	3.7 × 10^–7^
Mercury	cg03085637	*GNRHR2*;	1	145,918,458	Linear	1.72	0.33	1.3 × 10^–7^
		*PEX11B*			MOA	0.04	0.01	1.6 × 10^–7^
	cg16845679	N/A	12	114,624,854	Linear	− 0.85	0.16	2.0 × 10^–7^
					MOA	− 0.04	0.01	2.3 × 10^–7^
Metallurgy	cg07503918	*PRKG1-AS1*	10	52,308,834	Linear	1.39	0.25	1.8 × 10^–8^
					MOA	0.05	0.01	3.1 × 10^–8^
	cg10071091^2^	*KSR2*	12	117,762,563	Linear	− 1.57	0.31	3.0 × 10^–7^
					MOA	− 0.05	0.01	4.4 × 10^–7^

^1^MOA and linear not on the same scale.

^2^Probe is not methylome-wide significant for the MOA model, but is for the linear model.

**Figure 3:**
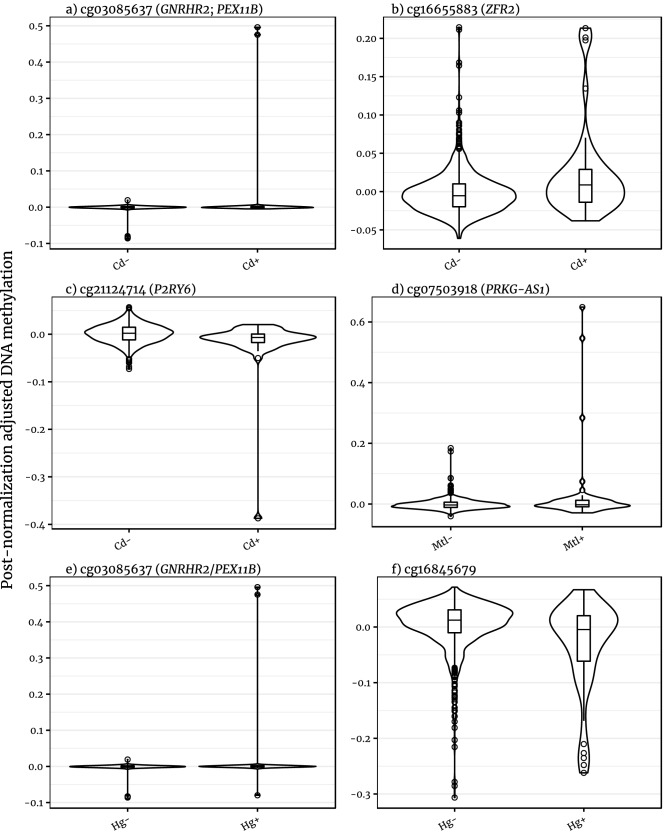
Violin plots of the distribution of normalised DNA methylation values for significant probe-exposure associations. Cd: cadmium; Mtl: metallurgy; Hg: mercury; -: no exposure + : exposure.

The direction of differential methylation at these CpG sites was mixed (Fig. 3, Table 2). In response to cadmium exposure, cg03085637 (in proximity to *GNRHR2* and within *PEX11B*) and cg16655883 (annotated to *ZFR2*) were hypermethylated, while cg21124714 (*P2R76*) was hypomethylated. For Metallurgy, cg07503918 (*PRKG1-AS1*) was hypermethylated and the cg10071091 site hypomethylated. Mercury exposure replicated a similar effect for cg03085637 as cadmium exposure, while cg16845679 (an intergenic CpG site on on chromosome 12) was hypomethylated.

The association of the cg03085637 site to both cadmium and mercury exposure highlights the correlation between the two exposures and abnormal hypermethylation. For example, of 855 participants, 19 reported exposure to cadmium and not mercury, 30 to mercury and not cadmium, and 21 to both. Figure 3 shows the DNAm values for these epigenome-wide significant sites. Figure 3a shows cg03085637 DNAm values plotted by the cadmium exposure, while Fig. 3e shows the sites separated by the mercury exposure. Notably, the significance of this *GNRHR2*/*PEX11B* probe is driven by two cadmium and mercury-exposed individuals with extreme hypermethylation ($v > {\text{Q}}_{3} + 100 \times {\text{IQR}}$, where IQR is the interquantile range, Q is the quantile and $v$ is the adjusted methylation value), which illustrates the difficulty in differentiating the exposures.

This pattern of few extreme methylation values skewing the distributions in the exposure groups is reflected in other MOA-significant DNAm sites. The association between DNAm values of cg21124714 and exposure to cadmium (Fig. 3c) was driven by one individual ($v \approx {\text{Q}}_{1} - 15 \times {\text{IQR}}$). Of the 22 individuals reporting having worked with metals, 6 showed extreme methylation in the cg07503918 site, as evident in Fig. 3d ($v \le {\text{Q}}_{3} + 9 \times {\text{IQR}}$). Both cadmium and mercury exposures are associated with DNAm levels which are not principally driven by a few outliers, cg16655883 and cg16845679, respectively (Fig. 3b,f). Associations at individual CpG sites were not supported by differences at nearby methylation loci, although this is common in MWAS due to lack of correlation between co-located probes on the array^35^.

Figures S9 and S10 show results of the permutation analyses of the MOA models for cadmium, mercury and metallurgical exposures. The six epigenome-wide significant loci remain significant under permutation mode as visualized in Figure S10. This indicates that the reported associations based on hyper- or hypomethylated outliers are unlikely to be incidentally captured by chance. However, genome-wide permutations given in Figure S11 show the number of epigenome-wide significant loci in this study was not more than expected by chance. Hence, these results should be treated with caution until future studies are able to provide validation data.

Our DMR analysis of the MOA results indicate that none of the previously detected significant probes fall within DMRs and exist as singular hyper- or hypomethylated points. However, the DMR analyses were conducted on the subset of probes passing the 0.02 SD threshold on the already limited 450 k probe array which covers a small fraction of CpG sites across the genome. Figure S12 is a histogram displaying the number of probes in a given 750 bp window, with 43.4% of the probes in the analysis having no others within their own window (Figure S12a). There is little evidence of artificial inflation of the previous results due to spatially correlated probes; however, this does not discount that methylation can occur in the flanking region. Of the 15 phenotypes tested through comb-p, 13 reported DMRs of more than 1 probe with significant Sidak-corrected regional p-values, with the highest number ($n = 16$) of DMRs associated with bore water exposure. All DMRs comprised probes with the same direction of effect. These significant DMRs are reported for each tested phenotype in the supplementary file Table S4.

## Discussion

We performed MWAS with a range of environmental exposures potentially associated with ALS, identifying several associations between DNAm and exposure to heavy metals in analyses that fitted ALS case/control status as a covariate. DNAm near the *PEX11B* gene was found to be associated with cadmium exposure. *PEX11B* is expressed widely across tissue types, and its aggregation on the perixosomal membrane initiates the elongation and then constriction required in the replication pathway of peroxisomes^36^. This gene family has been measured to respond to cadmium exposure in Arabidopsis in targeted time-course analyses, with increased expression of *PEX11* isoforms (*PEX11A* and *PEX11E*)^37^.

We replicated previously published observations of methylation changes in response to smoking status. Unlike smoking, the effects of the other exposures on DNAm were more limited, possibly attributable to reporting and recall. Exposures were variably reported, with cadmium exposure as the least frequent (5%). Exposures were self-reported or derived in the case of diesel, with severity, duration and temporal period unreported for all exposures excepting smoking. We acknowledge that exposures should be regarded as continuous and not binary as encoded in the MWAS analysis, specifically those which are able to be physiologically measured through established biomarkers. For example, the long half-life of 13.6 years of Cadmium in urine^38^ would enable it to be a tested as a continuous outcome variable in MWAS if measured in this cohort. Including biological verification of self-reported exposures would be an advantage for future study designs of the epigenetic effects for environmental factors. Additionally, while extreme variations in methylation values (epivariations) are principally associated with epigenetic remodelling, others are a secondary consequence of *cis-*linked DNA mutations that can be verified through targeted sequencing ^39^.

There are several possibilities for the differences in epigenome-wide significant sites of methylation as a result of environmental exposures. In particular, cigarette smoke is a ubiquitous and highly frequent exposure with evidence of bioaccumulation in most of the population^40^ and highly associated tobacco-related biomarkers in active smokers^41^. Biomarkers for other exposures may have short half-lives in the span of weeks or be rapidly metabolized, as well as having low chemical concentrations, vulnerability to handling contamination^42^, and variability in measurements within and between laboratories^43^. Measurable toxicants emitted per cigarette dose are comparatively large, with tar levels between $7.4 - 19.4$ mg^44^. In comparison, ambient air cadmium concentrations measured at a cadmium pigment factory ranged from $0.02 - 1.22$ mg/m^3^ in the first year, to $0.01 - 0.52$ mg/m^3^ in the second, depending on the tasks performed^45^. Finally, the measurement method (self-reported exposure as a binary trait) limits the detection of the effect. Our permutation analyses highlighted that while the individual significant probes have robust significance, we did not observe more significant probes epigenome-wide than expected by chance. These limitations indicate the need for substantially larger sample sizes to robustly detect associations with such exposures.

Correlation between exposures reported by participants introduces additional difficulties in interpreting the relationship between DNAm and exposures. This is illustrated by the association with *PEX11B* and cadmium, where the two individuals who drove the significance of this effect both shared self-reported cadmium and mercury exposures. Whether this effect is related to either of the two reported metal-based variables, or to another unknown shared exposure or cause remains indecipherable with the available data and the limitation of it being observed in only two individuals. Replication in other data sets is needed to verify these results. The first principal component of all metal-related exposures, which accounted for 51% of their variance, was used as a proxy for this invisible “lifestyle type”. However, no associations were identified with metal-PC1 at the methylome-wide significance threshold.

In summary, we attempted to predict a set of self-reported environmental exposures through an MWAS consisting of 855 ALS cases and controls. We illustrate the utility of capturing historical exposures as a way of elucidating the epigenome, with the qualifier that larger study cohorts will be required to differentiate these effects. While smoking may be detected in a small cohort owing to its effect size, other exposures will require more individuals to distinguish small effects or to correctly differentiate outliers in methylation. We present the summary statistics of associations to allow future replication and meta-analyses.

## Supplementary Information

Supplementary Information 1.

Supplementary Information 2.

## Data Availability

The DNA methylation array data are available in the NCBI dbGaP repository with accession number phs002068.v1.p1.
