# Supplementary material for: Association between DNA methylation variability and self-reported exposure to heavy metals
Source: Sci Rep. 2022 Jun 22;12:10582. doi: 10.1038/s41598-022-13892-w (PMC9217962; doi:10.1038/s41598-022-13892-w)
Supplement: Supplementary file 1 — Supplementary Information 1. [file 41598_2022_13892_MOESM1_ESM.docx]

**Supplementary Materials**

**Association between DNA methylation variability and self-reported exposure to heavy metals**

Anna Freydenzon^1^, Marta Nabais^1,2^, Tian Lin^1^, Kelly L Williams^3^, Leanne Wallace^1^, Anjali K Henders^1^, Ian P Blair^3^, Naomi R. Wray^1,4^, Roger Pamphlett^5^, Allan F. McRae^1^^

^1^ Institute for Molecular Bioscience, The University of Queensland, Brisbane, QLD 4072, Australia

^2^ University of Exeter Medical School, Exeter EX2 5DW, Devon, UK

^3^ Centre for Motor Neuron Disease Research, Macquarie University, NSW 2109, Australia

^4^ Queensland Brain Institute, The University of Queensland, Brisbane, QLD 4072, Australia

^5^ Brain and Mind Centre, The University of Sydney, Sydney, NSW 2050, Australia

^^^ Corresponding author: [a.mcrae@uq.edu.au](http://a.mcrae@uq.edu.au)

**
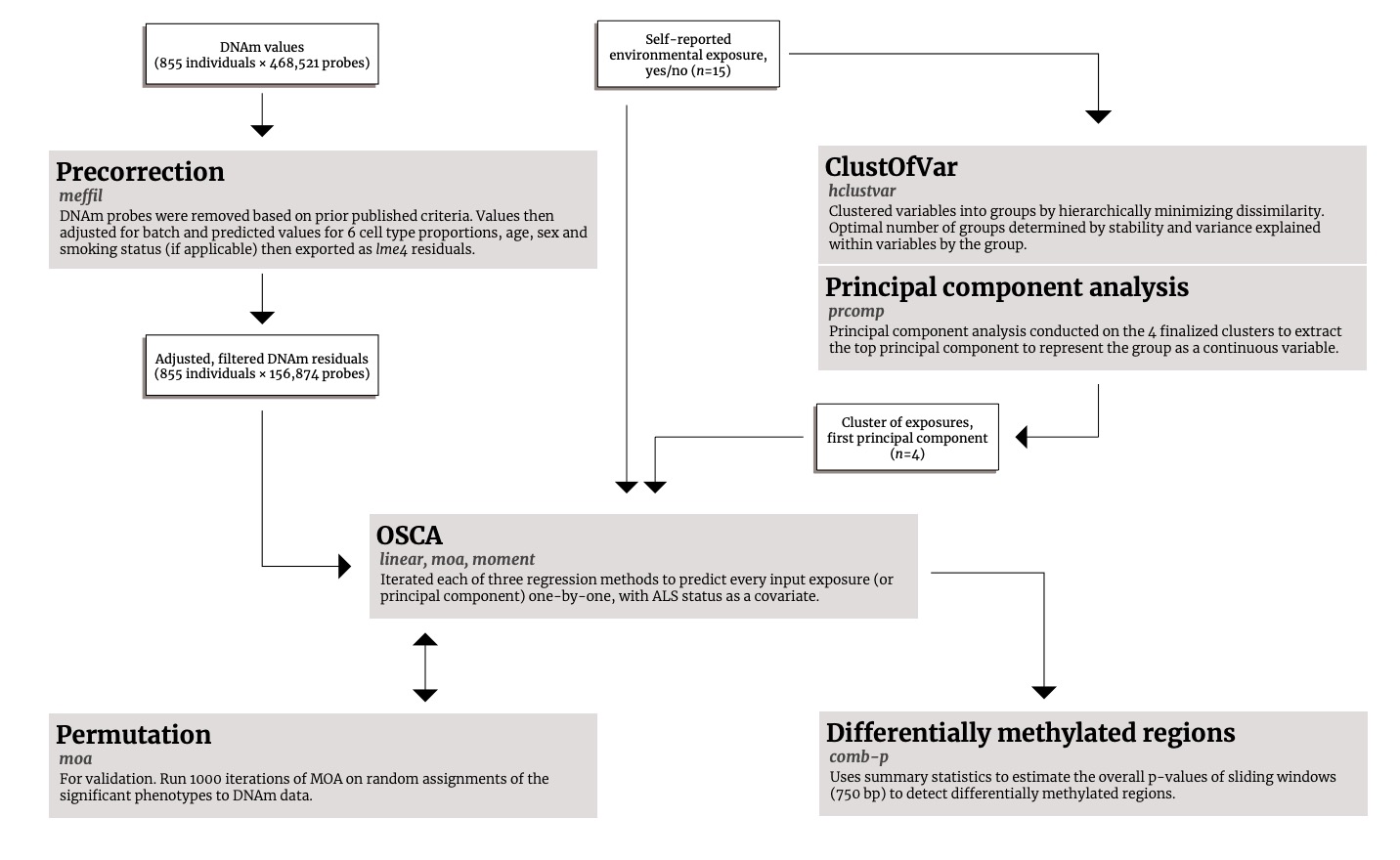
Supplementary Figure S1.** Overview of the methylation-wide association study (MWAS) workflow. Steps are detailed with the software used and its functionality, as well as intermediate outputs.

**Supplementary Figure S2**. Stability of hierarchical cluster partitions across 100 sampling bootstraps. The annotated star indicates the final chosen cluster count ($k = 4)$. Cluster stability was used as a metric for deciding on the number of partitions based on the integrity of the clusters to random sampling, which drops from $93.2\%$ at$k = 4$to$79.3\%$ at $k = 5.$

**Supplementary Figure S3**. Variance explained by the first principal component across each cluster partitioning up to *k* = 4 clusters. Bars in the selected *k* = 4 are coloured to the final selected groupings containing related variables: Blue for Mine, green for Farm, brown for Chem and purple for Work.

**Supplementary Figure S4**. Principal component analyses on finalized environmental variable clusters with projected factor loadings.

**Supplementary Figure S5**. Distribution of normalised DNA methylation values of the three MOMENT-significant probes for smoking, by smoking status and disease group.

**Supplementary Figure S6**. QQ plot of epigenome-wide association ($-log{10}_{p}$) between normalised DNA methylation measures and current smoking status across linear, MOA and MOMENT models.

**Supplementary Figure S7**. Epigenome-wide association ($-log{10}_{p}$) between normalised DNA methylation measures and current smoking status across linear, MOA and MOMENT models.

**Supplementary Figure S8**. Epigenome-wide association ($-log{10}_{p}$) between normalised DNA methylation measures and environmental exposures in MOA models.

**Supplementary Figure S9.** Q-Q plots contrasting MOA and linear-model$-log{10}_{p}$ of association between normalised DNA methylation measures and environmental exposures, excluding smoking.

**Supplementary Figure S10**. Density plot of -log10 transformed p-values for identified significant associations across 1000 permutations of the phenotypes tested by the MOA model for the six signficantly associated probes. For the probe DNAm values the exposure phenotype is randomly reallocated across people in each permuation. The dashed line with the annotated label designates the unpermutated transformed p-value for that probe.

**Supplementary Figure S11**. Histogram of the number of significant probes detected across 1000 permutations of the phenotypes tested under the MOA model applied genomewide. The dashed line designated the unpermuted number of probes previously detected for that phenotype.

**Supplementary Figure S12**. Histogram of probes containing $n$ other probes within the flanking ±750 bp window, striated by the analysis-filtered or full subset of probes passing QC in the array.

**Supplementary Table S1**. Distribution of years of age at collection time between ALS cases and controls.$Q(p)$ is the quantile of ages under $p$ proportion.

**Supplementary Table S2**. ANZCO and ISCO occupation codes interpreted as having likely diesel exposure.

**Supplementary Table S3**. Top 20 DNA methylation probes associated with current self-reported smoking status in the MOA model.
